# Prognostic value of arterial carbon dioxide tension during cardiopulmonary resuscitation in out-of-hospital cardiac arrest patients receiving extracorporeal resuscitation

**DOI:** 10.1186/s13049-024-01195-0

**Published:** 2024-03-21

**Authors:** Pei-I Su, Min-Shan Tsai, Wei-Ting Chen, Chih-Hung Wang, Wei-Tien Chang, Matthew Huei-Ming Ma, Wen-Jone Chen, Chien-Hua Huang, Yih-Sharng Chen

**Affiliations:** 1 Department of Emergency Medicine, National Taiwan University Hospital and National Taiwan University College of Medicine, National Taiwan University, No.7, Zhongshan S. Rd., Zhongzheng Dist., Taipei City, 100 Taiwan (ROC); 2 Departments of Internal Medicine, National Taiwan University Hospital and National Taiwan University College of Medicine, Taipei, Taiwan; 3 Department of Surgery, National Taiwan University Hospital, and College of Medicine, National Taiwan University, Taipei, Taiwan

**Keywords:** Extracorporeal cardiopulmonary resuscitation (ECPR), Out-of-hospital cardiac arrest (OHCA), Carbon dioxide tension (PaCO2), Neurological outcome

## Abstract

**Background:**

Current guidelines on extracorporeal cardiopulmonary resuscitation (ECPR) recommend careful patient selection, but precise criteria are lacking. Arterial carbon dioxide tension (PaCO_2_) has prognostic value in out-of-hospital cardiac arrest (OHCA) patients but has been less studied in patients receiving ECPR. We studied the relationship between PaCO_2_ during cardiopulmonary resuscitation (CPR) and neurological outcomes of OHCA patients receiving ECPR and tested whether PaCO_2_ could help ECPR selection.

**Methods:**

This single-centre retrospective study enrolled 152 OHCA patients who received ECPR between January 2012 and December 2020. Favorable neurological outcome (FO) at discharge was the primary outcome. We used multivariable logistic regression to determine the independent variables for FO and generalised additive model (GAM) to determine the relationship between PaCO_2_ and FO. Subgroup analyses were performed to test discriminative ability of PaCO_2_ in subgroups of OHCA patients.

**Results:**

Multivariable logistic regression showed that PaCO_2_ was independently associated with FO after adjusting for other favorable resuscitation characteristics (Odds ratio [OR] 0.23, 95% Confidence Interval [CI] 0.08–0.66, *p*-value = 0.006). GAM showed a near-linear reverse relationship between PaCO_2_ and FO. PaCO_2_ < 70 mmHg was the cutoff point for predicting FO. PaCO_2_ also had prognostic value in patients with less favorable characteristics, including non-shockable rhythm (OR, 3.78) or low flow time > 60 min (OR, 4.66).

**Conclusion:**

PaCO_2_ before ECMO implementation had prognostic value for neurological outcomes in OHCA patients. Patients with PaCO_2_ < 70 mmHg had higher possibility of FO, even in those with non-shockable rhythm or longer low-flow duration. PaCO_2_ could serve as an ECPR selection criterion.

**Supplementary Information:**

The online version contains supplementary material available at 10.1186/s13049-024-01195-0.

## Background

Current guidelines on ECPR recommend careful selection of patients with OHCA with potential reversible causes and limited periods, but the precise criteria are lacking [1]. ECPR activation protocols vary across different institutes with discrepant outcomes [2]. Previous studies showed that certain characteristics were associated with favorable neurological outcome (FO) in patients with OHCA receiving extra-corporeal cardiopulmonary resuscitation (ECPR), including witnessed arrest, shockable cardiac rhythm, and shorter low flow time [3–8]. Most protocols relied on the presence of shockable cardiac rhythm and short time period before ECMO implementation. However, there were some important resuscitation characteristics that were hard to quantify, including no flow duration, quality of pre-hospital CPR, and physical reservoir of individuals. Recent studies reported that regional cerebral oxygen saturation have prognostic value during ECPR [9], but this technique warranted further resources and training. Blood sampling was easy to be obtained and interpreted. Metabolic parameters (blood pH, carbon dioxide tension, and lactic acid) have been demonstrated to have prognostic value in patients with OHCA [10, 11], but the results have been less studied in patients receiving ECPR. The metabolic derangement may help estimate the extent of hypoxic injury [4] and make ECPR selection more precisely.

During cardiac arrest, the decreased cardiac output state causes ventilation-perfusion mismatch by decreasing pulmonary perfusion, which decreases carbon dioxide (CO_2_) exchange. This leads to CO_2_ accumulation in tissues and blood. Hypercapnia also increases cerebral blood flow, with increased intracranial pressure and cerebral edema [12, 13]. Effective cardiopulmonary resuscitation (CPR) can decrease blood CO_2_ as demonstrated in previous studies [14–16]. PaCO_2_ level could serve as a marker for perfusion status during CPR [16]. We hypothesised that the PaCO_2_ obtained during cardiac arrest could have prognostic value in OHCA patients receiving ECPR and could guide ECPR selection. We aimed to study the relationship between PaCO_2_ and outcomes of patients with OHCA receiving ECPR.

## Methods

### Study setting and patient inclusion

This retrospective study was conducted in a tertiary hospital in Taipei City, Taiwan, with an emergency department with more than 1,15,000 visits annually and 220 intensive care unit (ICU) beds.

This study included patients with OHCA aged ≥ 18 years who were treated with ECPR between January 2012 and December 2020. Patients were excluded for the following reasons: 1) ECPR was initiated at another hospital and the patient was transferred after ECMO, 2) traumatic OHCA, and 3) OHCA with sustained ROSC (ROSC for more than 20 min), but ECMO was warranted for haemodynamic support.

### ECPR criteria and ECMO bundle care

The prehospital EMS system in the OHCA setting was reported in a previous study [17]. There were basic life support teams with defibrillation ability and advanced life support teams, which are qualified for intubation and intravenous epinephrine injection in the prehospital setting.

Advanced airway was established for 60 percent of patients. Chest compressions were performed with mechanical CPR devices for 85 percent of patients during transport, unless not feasible because of body size or other reasons. The dispatch center would inform the emergency department by telephone of the incoming OHCA patient information, including patient`s age, gender, prehospital DC shock times, prehospital intervention, and estimated arrival time.

If OHCA patients did not achieve ROSC after 10 min of standard Advanced Cardiac Life Support (ACLS), the emergency physician would discuss with the duty cardiovascular surgeon for ECPR eligibility. Patients were considered eligible for ECPR if they met all the following criteria: (1) age < 80 years, (2) witnessed collapse with no-flow time < 10 min, (3) pre-disease cerebral performance category (CPC) of 1–2 and no terminal malignancy, (if CPC category was not available, the pre-disease neurological status, as Glascow Coma Scale, would be used instead. All the eligible patients must have a GCS score of 15) and (4) presumed reversible cause (e.g. acute coronary syndrome or pulmonary embolism). The cardiovascular surgeon made the final decision on ECMO eligibility. The emergency department had a routine team structure while managing patients with OHCA. One nurse focused on blood sampling through puncturing femoral artery and the other nurse obtained venous access and epinephrine injection. The blood gas analysis was performed right after blood sampling and sent for point-of-care analysis using SIEMEMS RAPIDPoint 500 Systems in the resuscitation room. Arterial pH, PaCO2, and lactic acid levels were interpreted to guide further resuscitation.

The ECMO cannulation approach in our hospital is peripheral cannulation with open technique. Cannulation was performed under direct vision of femoral vessels with modified Seldinger method. All the cannulations were performed in the emergency department by the duty cardiovascular surgeon with the assistance of ECMO technicians. The ECMO components included a centrifugal pump and oxygenator (Medtronic, Anaheim, CA; Medos, Stolberg, Germany; Maquet, Rastatt, Germany). After ECMO, the patient underwent computed tomography to survey for possible OHCA causes, and the cardiologist evaluated the feasibility of coronary angiography. The patient was admitted to the ICU for post-resuscitation care.

### Data collection

Baseline characteristics and comorbidities were recorded in the medical records and retrospectively collected. Resuscitation variables were collected from the emergency medical service and hospital records. All time intervals were retrospectively calculated from the hospital records. Arrest-hospital time was defined as the time interval between cardiac arrest (CA) and hospital arrival. The arrest-ECMO time was defined as the time from CA to ECMO implementation. Arterial pH, PaCO_2_, and lactic acid levels were recorded from the first blood sample at the emergency department. Data on the intervention after ECPR, survival, and neurological outcomes at discharge were collected from the medical records.

### Outcome

The primary outcome was a favorable neurological outcome at discharge, defined as a Cerebral Performance Category score of 1 (good cerebral performance) or 2 (moderate cerebral disability). The secondary outcome was survival until hospital discharge.

### Statistics

Categorical variables are expressed as percentages and were compared using the chi-squared test. Continuous variables are expressed as means ± standard deviations, and t-tests were used to delineate differences. Statistical significance was set at *p*-value < 0.05. The predictive abilities of metabolic parameters (pH, PaCO_2_, and lactic acid) were tested using the area under the receiver operating characteristic (ROC) curve (AUC). We used a generalised additive model (GAM) to determine the relationship between PaCO_2_, FO, and survival. All variables with a *p*-value < 0.15 were included in multivariable logistic regression to determine the independent variables for predicting FO. All the time variables were treated as continuous variables in the multivariable regression model. A stepwise backward elimination method was used to select the final regression model. The selected model was then compared with current prognostic scores (including the TIPS 65 score [18], OHCA score [19], TTM score [20], and rCAST score [21, 22]). Subgroup analyses were performed to test the discriminative ability of PaCO_2_ in different subgroups: initial shockable cardiac rhythm versus non-shockable rhythm, hospital arrival time < 25 min versus > 25 min, and arrest-ECMO time < 60 min versus > 60 min. The p-value for the interaction was tested using an interaction test. All computations were performed using SPSS, version 16.0 (IBM Corp., Armonk, NY, USA).

## Results

### The characteristics of study population

**Fig. 1:**
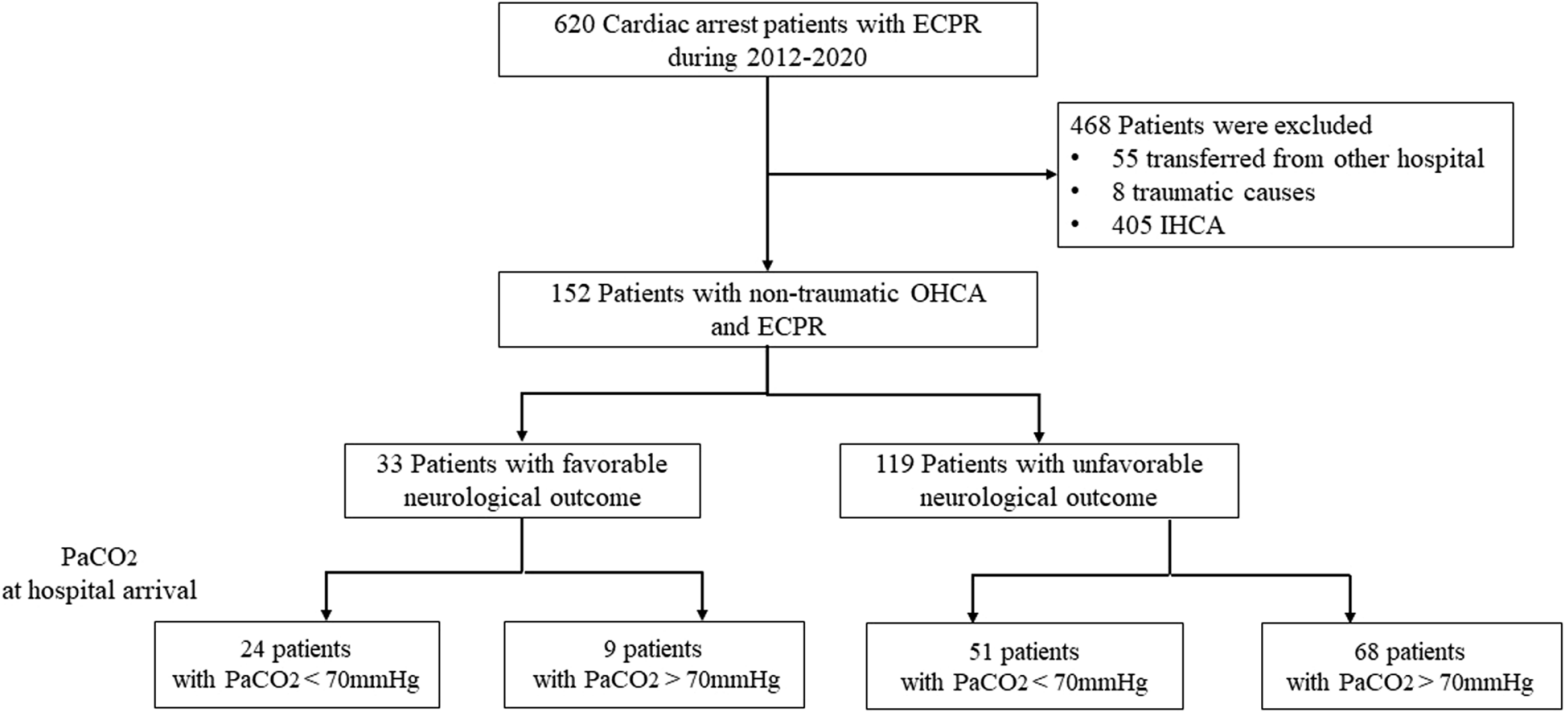
Patient enrolment

Between January 2012 and December 2020, 152 OHCA patients who received ECPR were included (Fig. 1). FO at discharge was noted in 21% of the patients (33/152) and survival to discharge in 34% (52/152). The characteristics of the patients who received ECPR are summarised in Table 1 and stratified according to neurological outcomes and survival at discharge. The median age was 55 years, and the patients were predominantly male. Cardiac origin was noted in 68% of the patients (62% with acute coronary syndrome, 5% with fatal arrhythmia or cardiomyopathy, and 1% with acute myocarditis). Shockable cardiac rhythm was noted in 75% of the patients. The mean arrest-ECMO time was 59.6 min. Patients with FO were more likely to have witnessed arrest, shockable cardiac rhythm, shorter hospital arrival time, and shorter arrest-extracorporeal membrane oxygenation (ECMO) time. For the metabolic factors, patients with FO had lower pH value and PaCO_2_ (56.7 mmHg versus 71.1 mmHg, *p* = 0.007), while lactic acid level showed no difference.

**Table 1:** The characteristics of patients with ECPR stratified according to neurological outcome and survival at discharge

	All Patients (*n* = 152)	Favorable neurological outcome (*n* = 33)	Non-favorable neurological outcome (*n* = 119)	*p*-value	Survivor (*n* = 52)	Non-survivor (*n* = 100)	*p*-value
Gender(male) (%)	132 (86.8)	26(78.8)	106(89.1)	0.209	44(84.6)	88(88.0)	0.739
Age (SD)	55.5(11.4)	55.4(12.0)	55.3(11.3)	0.962	55.0(11.1)	55.4(11.6)	0.823
**Comorbidity**							
CAD (%)	43 (28.2)	8(24.2)	35(29.4)	0.715	14(26.9)	29(29.0)	0.936
DM (%)	49 (32.2)	9(27.3)	40(33.6)	0.631	17(32.7)	32(32.0)	1
ESRD (%)	6 (3.9)	0(0)	6(5.0)	0.417	0(0)	6(6.0)	0.172
COPD (%)	3 (1.9)	1(3.0)	2(1.7)	1	1(1.9)	2(2.0)	1
**Resuscitation variables**							
Witnessed Arrest (%)	127 (81.9)	32(97.0)	95(79.8)	0.037	49(94.2)	78(78.0)	0.019
Bystander CPR (%)	109 (71.7)	28(84.8)	81(68.1)	0.093	40(76.9)	69(69.0)	0.401
Location (public) (%)	102 (67.1)	27(81.8)	75(63.0)	0.068	40(76.9)	62(62.0)	0.093
Initial shockable (%)	115 (75.7)	26(78.8)	89(74.8)	0.807	42(80.8)	73(73.0)	0.39
Shockable at ER (%)	75 (49.3)	23(69.7)	52(43.7)	0.014	33(63.5)	42(42.0)	0.019
Changing rhythm (%)	57 (37.5)	17(51.5)	40(33.6)	0.093	25(48.1)	32(32.0)	0.077
No flow time (SD)	3.28 (5.3)	1.91(1.5)	4.30(5.6)	< 0.0001	3.42(4.4)	3.96(5.4)	0.512
Arrest -hospital time (SD)	25.8 (15.4)	21.5(10.6)	27.0(16.3)	0.026	24.9(13.2)	26.2(16.4)	0.632
Arrest -ECMO time (SD)	65.8 (20.4)	57.6(13.1)	68.7(21.3)	0.0004	63.0(18.9)	68.0(20.9)	0.143
**Metabolic factor**							
pH (SD)	7.02 (0.15)	7.077(0.15)	7.01(0.15)	0.047	7.06(0.16)	7.01(0.15)	0.099
Lactate acid (SD)	11.68 (4.4)	11.71(4.9)	11.68(4.3)	0.975	11.77(4.8)	11.64(4.2)	0.867
PaCO2 (SD)	68.1 (26.2)	56.7(24.9)	71.2(25.8)	0.007	61.1(26.8)	71.7(25.3)	0.026
**Intervention**							
CAG (%)	110 (72.3)	33 (100)	77 (64.7)	0.0006	52 (100)	58 (58)	0.004
Arrest-CAG time (SD)	159.3 (60.0)	148.1 (67.2)	165.0 (58.1)	0.354	149.2 (61.4)	170.5 (61)	0.241
TTM (%)	78 (51.3)	13 (39.3)	65 (54.6)	0.111	25 (48.0)	53 (53.0)	0.524

*CAG* coronary angiography, *TTM* Targeted temperature management

### The predictive value of PCO_2_ compared to other metabolic factors

**Fig. 2:**
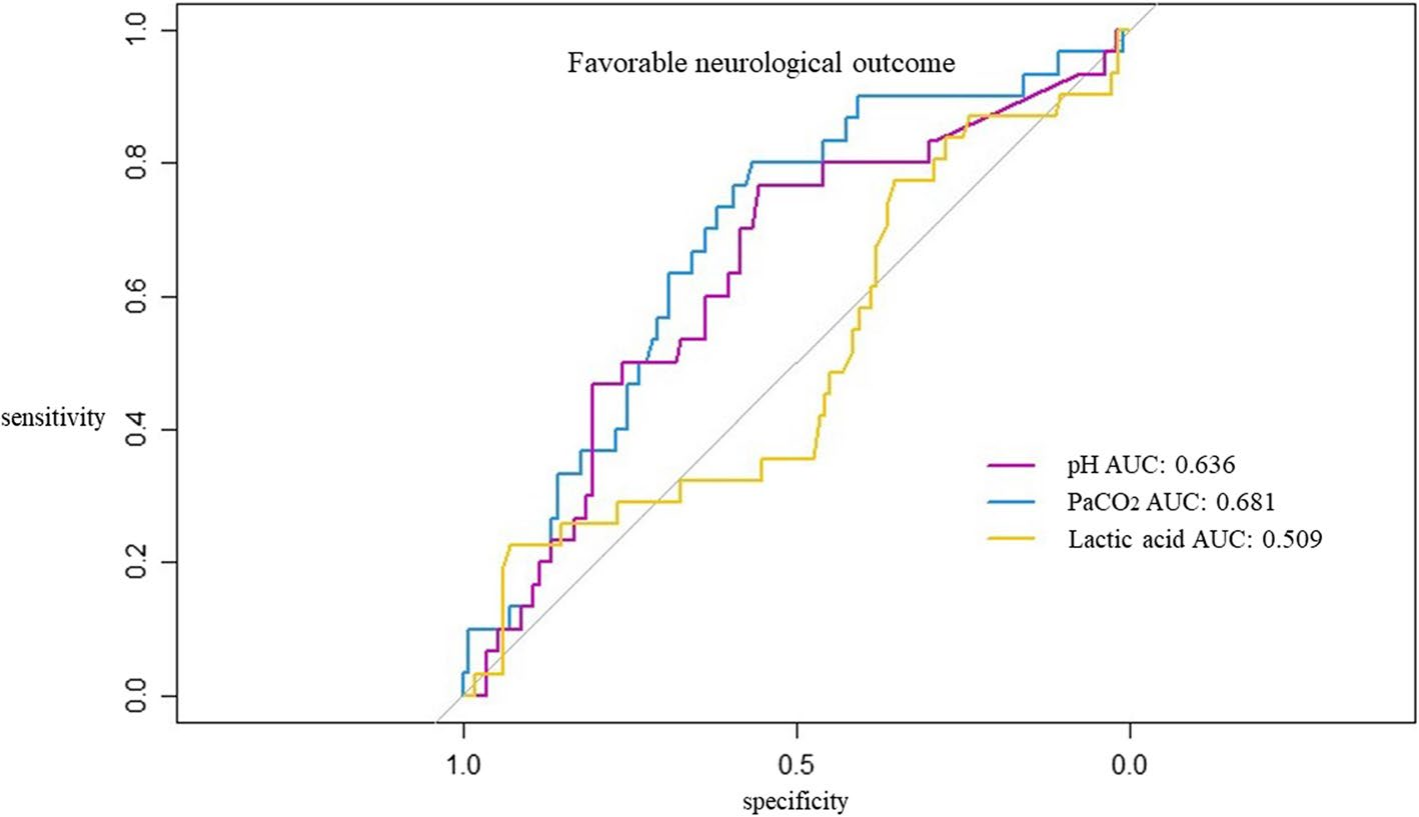
The receiver operating characteristic curve (ROC) and the area under curve (AUC) of Arterial carbon dioxide tension (PaCO_2_), pH, and lactic acid to favorable neurological outcome at discharge

The ROC curve and AUC of PaCO_2_, pH, and lactic acid for predicting FO are shown in Fig. 2. PaCO_2_ had the highest AUC of 0.681 (95% CI, 0.576–0.786, *p* = 0.002).

**Fig. 3:**
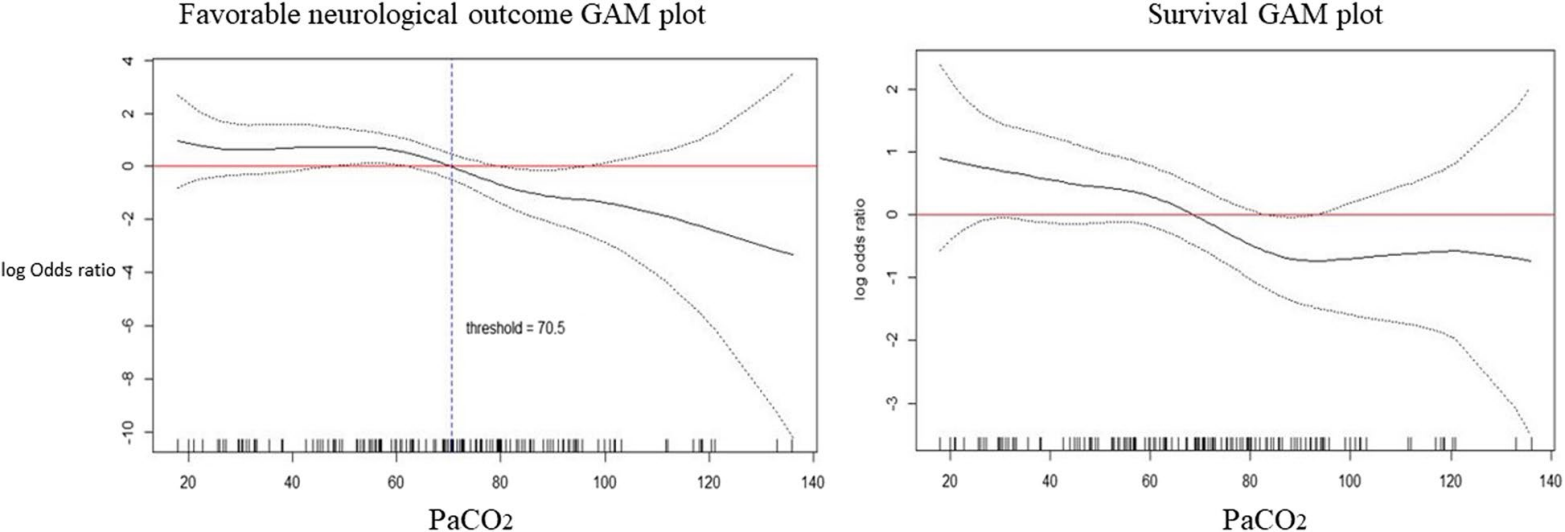
The near-linear reverse relationship of PaCO_2_ with favorable neurological outcome (FO) and survival demonstrated by generalized additive model. PaCO_2_ 70 mmHg was noted as the cut point for differentiating patients with favorable neurological outcome

We used GAM to test the relationship between PaCO_2_, FO, and survival, and the results showed a near-linear reverse relationship (Fig. 3). PaCO_2_ 70 mmHg was noted as the cutoff point for differentiating FO from non-FO patients, and PaCO_2_ 63 mmHg was the cutoff point for survival. PaCO_2_ about 70 mmHg also had the highest Youden index in the ROC curve (Supplementary Table 1: Youden index).

### Independent predictors for favorable neurological outcome and survival

Multivariate logistic regression showed that PaCO_2_ < 70 mmHg was independently associated with FO after adjusting for other favorable resuscitation characteristics (Table 2). The final regression model with the highest discriminant ability contained six independent resuscitation variables: no flow duration, PaCO_2_ < 70 mmHg, witnessed arrest, rhythm change to non-shockable before ECMO, shockable rhythm at hospital arrival, and arrest at a public location. The AUC of the regression model was higher than that of the other current scores for predicting the neurological outcome prediction (Supplementary Fig. 1).

**Table 2:** Multivariable logistic regression to favorable neurological outcome of variables with *p*-value < 0.15. The “No flow time” and “Arrest-ECMO time” were treated as continuous variables

Variable	Crude Odds Ratio	Adjusted Odds Ratio	95% C.I	*p*-value
No flow time	0.85	0.80	0.63—1.02	0.072
Arrest-ECMO time	0.96	0.99	0.97—1.02	0.678
PaCO2 (> 70 mmHg)	0.19	0.23	0.08—0.66	0.006
Witnessed Arrest	8.08	5.88	0.67—51.87	0.111
Rhythm change before ECMO	2.04	2.07	0.85—5.04	0.110
Shockable rhythm at hospital	2.96	2.05	0.79—5.27	0.138
Arrest location (at home)	2.47	2.62	0.88—7.79	0.084

### The correlation of PaCO_2_ with other resuscitation character

To study the correlation between PaCO_2_ and other resuscitation characteristics, we used a PaCO_2_ of 70 mmHg to separate the patients into two groups (Table 3). Patients with a PCO_2_ < 70 mmHg had significantly shorter arrest-hospital and arrest-ECMO times. No differences were noted among other favorable characteristics, including no flow time, percentage of witnessed arrest, bystander CPR, public location arrest, and shockable rhythm.

**Table 3:** Correlation between PaCO_2_ and other resuscitation characteristics, stratified with PaCO_2_ 70 mmHg

Variable	PaCO_2_ < 70 (*n* = 75)	PaCO_2_ > 70 (*n* = 77)	*p*-value
Witnessed Arrest (%)	64(85.3)	57(74.0)	0.803
Bystander CPR (%)	54(72.0)	49(63.6)	1
location(public) (%)	55(73.3)	43(55.8)	0.263
Initial shockable (%)	53(70.7)	54(70.1)	0.312
Shockable at ER (%)	42(56.0)	27(35.0)	0.075
No flow time (SD)	3.743(5.263) (*n* = 74)	3.721(4.989)	0.977
Arrest to ER (SD)	21.37(14.72) (*n* = 71)	26.9(15.15)	0.0004
Arrest to ECMO (SD)	61.65(19.51)	72.28(20.04)	0.0017
pH (SD)	7.100(0.153)	6.945(0.098)	< 0.0001
Lactic acid (SD)	10.81(4.43) (*n* = 74)	12.62(4.32) (*n* = 67)	0.015
HCO3 (SD)	15.64(5.38)	18.97(6.81)	0.0016
K level (SD)	4.49(1.28) (*n* = 74)	4.42(1.10) (*n* = 67)	0.7000
Favorable neurological outcome (%)	24(32.0)	9(11.6)	0.0014
Survival (%)	32(42.7)	16(20.7)	0.0249

### Subgroup analysis

**Fig. 4:**
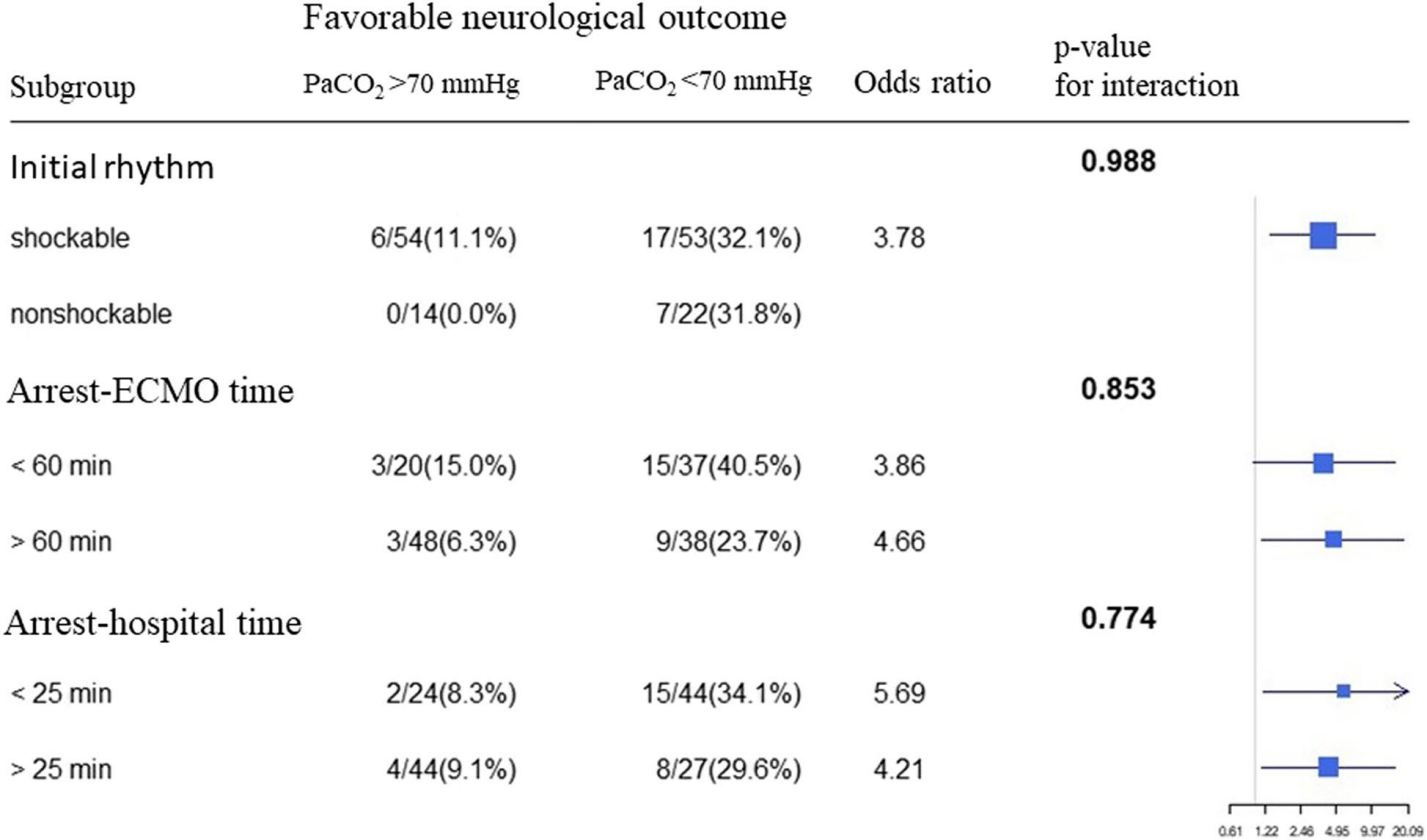
Subgroup analysis and interaction test for favorable neurological outcome according to initial cardiac rhythm, arrest- extracorporeal membrane oxygenation (ECMO) time, and arrest-hospital time

PaCO_2_ had prognostic value in different subgroups of patients (Fig. 4), including those with an initial non-shockable rhythm, longer hospital arrival time (OR = 4.21, in patients with hospital arrival time > 25 min), and longer ECMO implementation time (OR = 4.66, in those with ECMO time > 60 min). No subgroup showed a significant p value for the interaction.

## Discussion

In the present study, we demonstrated that PaCO_2_ during CPR is predictive of favorable neurological outcomes in patients with OHCA receiving ECPR and could improve ECPR selection.

Traditionally, ECPR was considered in patients with OHCA with witnessed arrest and possible cardiac origin (e.g. shockable rhythm). In our study, patients in the PaCO_2_ < 70 mmHg group had favorable resuscitation characteristics similar to those with higher PaCO_2_ but the FO percentage was significantly higher. This indicates that the PaCO_2_ level could be used as a powerful indicator in addition to traditional selection criteria. Combining PaCO_2_ with pre-specified criteria [5, 18, 23] (Supplementary Table 2) could further increase the percentage of favorable outcomes. For example, adding PaCO_2_ < 70 mmHg to the selection criteria in the CRITICAL study [23], the FO rate will improve from 19 to 33% with the exclusion of three patients with FO from our cohort.

Previous studies on the metabolic derangement of ECPR have focused on the pH and lactic acid level [24]. Okada et al. reported the influence of pH on FO of patients with OHCA receiving ECPR, with an AUC of 0.675 [25]. Our results demonstrated that PaCO_2_ had a higher discriminative value than pH or lactic acid. Only a few studies have examined the relationship between PaCO_2_ and neurological or survival outcomes after ECPR. Bartos et al. reported that PaCO_2_ increased as the flow time increased from 47 mmHg in 20 min to 70 mmHg in 90min [4], but the exact cut-off point for FO prediction has not been reported. Mandigers et al. reported that PaCO_2_ was not associated with neurological outcomes in the mixed 45% OHCA and 55% IHCA cohort [26] (PCO_2_ 53 mmHg in FO patients vs. 57 mmHg). These values are lower than those observed in our cohort. However, their PaCO_2_ was checked after ECMO when patients had already received advanced in-hospital management. The PaCO_2_ data in our study, using those checked shortly after hospital arrival could reflect more about the prehospital resuscitation status. To the best of our knowledge, our study is the first to report the prognostic value of PaCO_2_ and provide a cutoff point for ECPR selection.

PaCO_2_ increased with longer CPR duration [4], and one may argue that a lower PaCO_2_ simply reflects a shorter low flow duration and consequently a higher percentage of FO. However, even in patients with initial shockable rhythm and arrest-hospital time < 25 min, there was still a high heterogeneity of individual PaCO_2_ (Supplementary Fig. 2). Our hypothesis was that a lower PaCO_2_ level might be related to the better quality of prehospital CPR, adequate ventilation, and more physical reservoir of the individual. This could be reflected in the higher percentage of shockable rhythm at ED arrival, implying that these patients had better perfusion and therefore more viable myocardium to maintain shockable rhythm despite prolonged CPR. Since the prehospital CPR quality was difficult to quantify clearly, PaCO_2_ may be used to estimate the extent of ischaemia-hypoxic injury and help in ECPR selection. The arrest to blood-sampling time might also have influences on blood PaCO2 level. The distribution of arrest to blood-sampling time and correlation to arrest-hospital time were presented (Supplementary Fig. 3). The blood gas analysis was performed right after sampling with the instrument in the resuscitation room as routine in our hospital. Therefore, the arrest to blood-sampling time was almost identical to arrest-hospital time.

Shockable cardiac rhythm has a high predictability for cardiac origin and has been used as a selection criterion. Patients with a non-shockable rhythm had worse outcomes even with ECMO [27]. However, some ECPR criteria [2, 3] had no restriction on cardiac rhythm. Our results showed that PaCO_2_ also had prognostic value in patients with non-shockable rhythm or longer arrest-hospital arrival times. This indicated that PaCO_2_ could be a valuable factor when considering ECMO among those with less favorable characteristics, such as young patients with non-shockable rhythm or longer low-flow duration. Using PaCO_2_ as a selection could help identify those with the possibility of FO despite the lack of favorable characteristics. PaCO_2_ could serve as a useful adjuvant to different local ECPR policies.

### Clinical implication

The PaCO_2_ level before ECMO implementation had prognostic value for neurological outcomes among patients with OHCA. A PaCO_2_ level of 70 mmHg could help select patients with a higher possibility of favorable neurological outcomes, even among patients with non-shockable rhythm or longer low-flow duration.

### Limitation

This study had several limitations because of its retrospective nature. First, even though there were pre-specified ECPR initiation criteria in our hospital, the specific reason for initiating ECPR was based on the treating physician’s judgement. Some patients who may benefit from ECPR may have been skipped and not included. Second, the time records in this study were collected from electronic records. The exact no-flow time was difficult to calculate. Third, some patients had missing data on prehospital resuscitation variables, including EtCO_2_ and prehospital DC shock times. These data could provide additional information on the resuscitation status of patients. Fourth, some patients had extremely poor haemodynamic status even under ECMO, making the diagnosis or intervention infeasible. This could affect the estimation of the proportion of the cardiac origin.

### The correlation between arterial and venous CO_2_

Our PaCO_2_ samples were mostly obtained by direct puncture of the femoral artery during CPR. There might be incidental venous puncture due to difficulty in identifying pulsation, and the percentage of incidental venous puncture. Arterial and venous CO2 levels showed a good correlation. A previous study demonstrated that the mean difference in PCO_2_ was 4.8 mmHg with a Pearson correlation of 0.93, in critically ill patients [28]. However, the correlation was less studied in shock status or cardiac arrest [29].

## Conclusion

The PaCO_2_ level before ECMO implementation had prognostic value for neurological outcomes among patients with OHCA. Patients with PaCO_2_ < 70 mmHg had a higher possibility of FO, even among patients with non-shockable rhythm or longer low-flow duration. The PaCO_2_ level can serve as an ECPR selection criterion.

### Supplementary Information

**Additional file 1: Supplementary Fig. 1.** Comparison of the prediction ability of the final logistic regression model with current out-of-hospital cardiac arrest (OHCA) prediction models for neurological outcome.

**Additional file 2: Supplementary Fig. 2.** Distribution of PaCO_2_ level and arrest-hospital time according to neurological outcome.

**Additional file 3: Supplementary Fig. 3.** Distribution of arrest-blood sampling time and correlation to arrest-hospital time.

**Additional file 4. **

## Data Availability

The datasets used and analyzed during the current study are available from the corresponding author on reasonable request.

## Abbreviations

PaCO_2_: Arterial carbon dioxide tension
ED: Emergency department
