# Supplementary figures and images for: Prognostic value of arterial carbon dioxide tension during cardiopulmonary resuscitation in out-of-hospital cardiac arrest patients receiving extracorporeal resuscitation
Source: Scand J Trauma Resusc Emerg Med. 2024 Mar 21;32:23. doi: 10.1186/s13049-024-01195-0 (PMC10958860; doi:10.1186/s13049-024-01195-0)

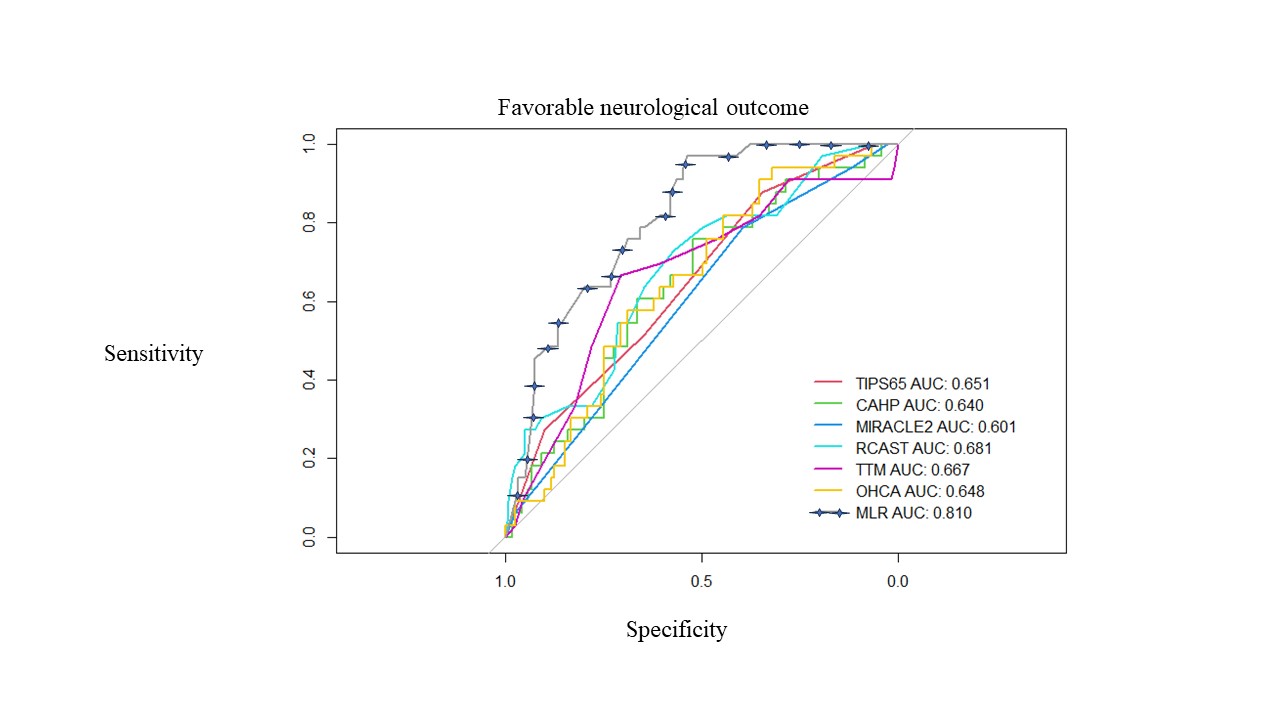

Supplement: Supplementary file 1 — Additional file 1: Supplementary Fig. 1. Comparison of the prediction ability of the final logistic regression model with current out-of-hospital cardiac arrest (OHCA) prediction models for neurological outcome. [file 13049_2024_1195_MOESM1_ESM.jpg]

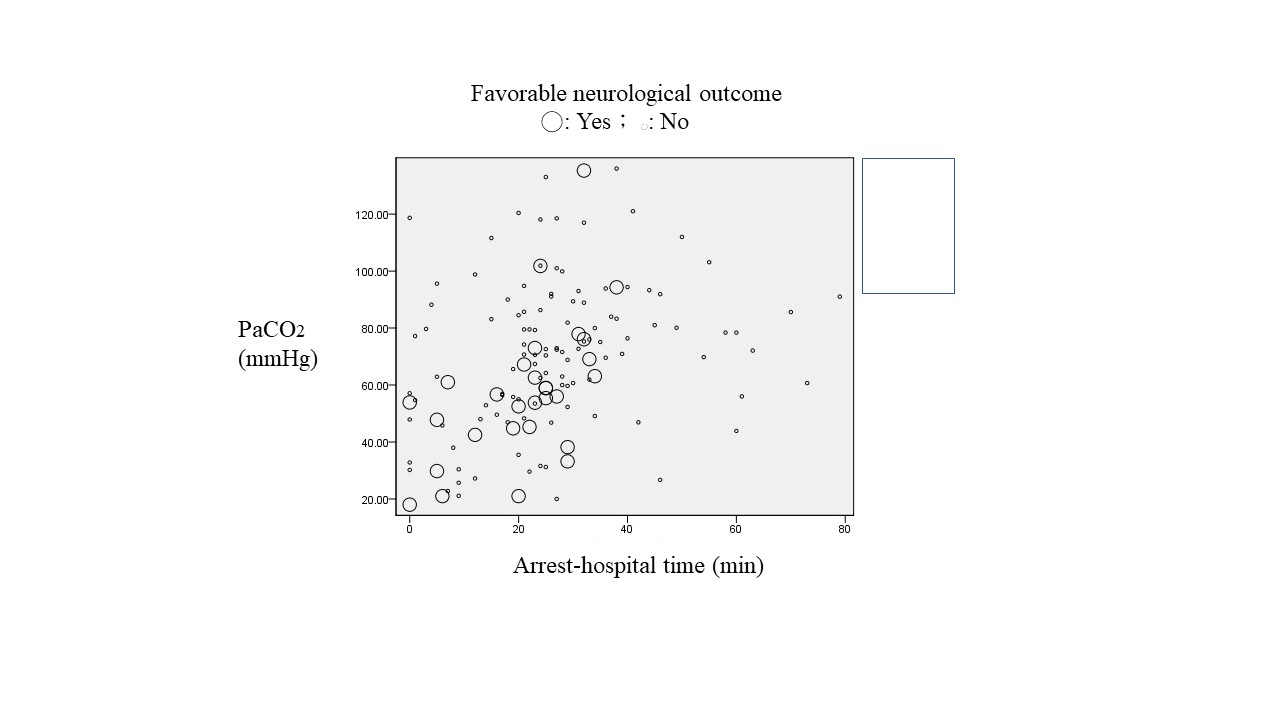

Supplement: Supplementary file 2 — Additional file 2: Supplementary Fig. 2. Distribution of PaCO2 level and arrest-hospital time according to neurological outcome. [file 13049_2024_1195_MOESM2_ESM.jpg]

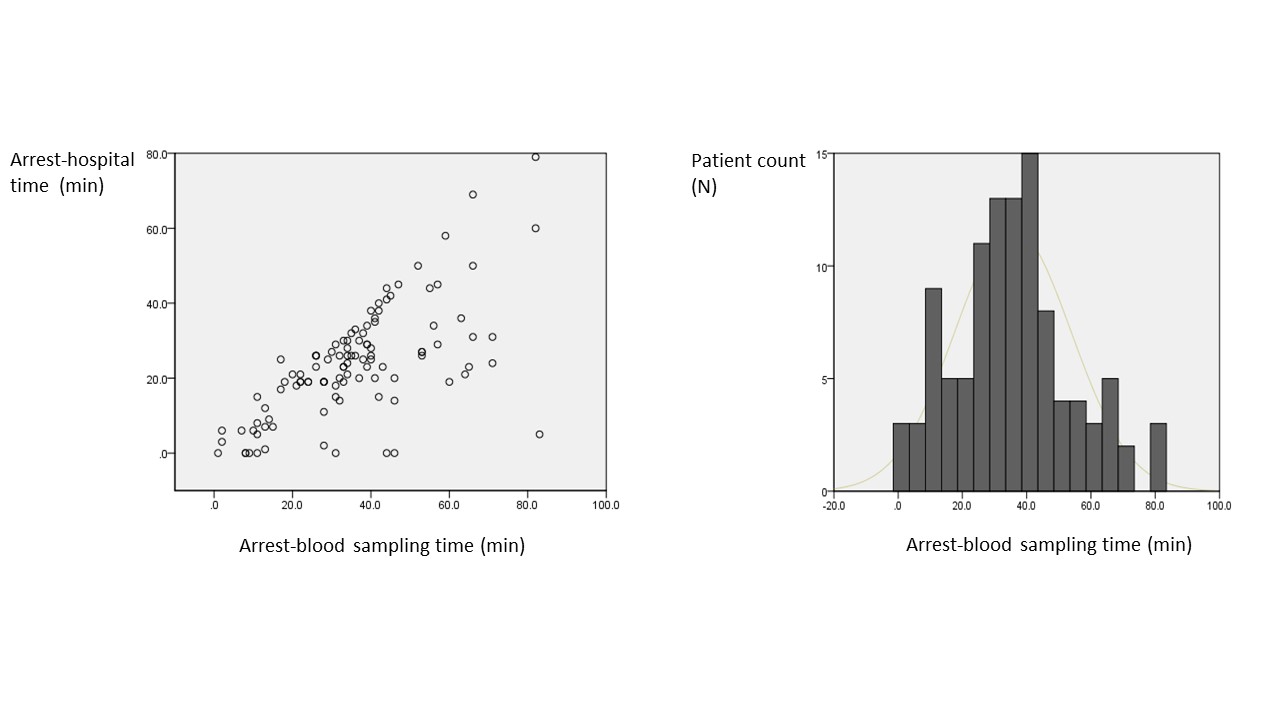

Supplement: Supplementary file 3 — Additional file 3: Supplementary Fig. 3. Distribution of arrest-blood sampling time and correlation to arrest-hospital time. [file 13049_2024_1195_MOESM3_ESM.jpg]
