# Supplementary material for: Prognostic value of arterial carbon dioxide tension during cardiopulmonary resuscitation in out-of-hospital cardiac arrest patients receiving extracorporeal resuscitation
Source: Scand J Trauma Resusc Emerg Med. 2024 Mar 21;32:23. doi: 10.1186/s13049-024-01195-0 (PMC10958860; doi:10.1186/s13049-024-01195-0)
Supplement: Supplementary file 4 — Additional file 4. [file 13049_2024_1195_MOESM4_ESM.docx]

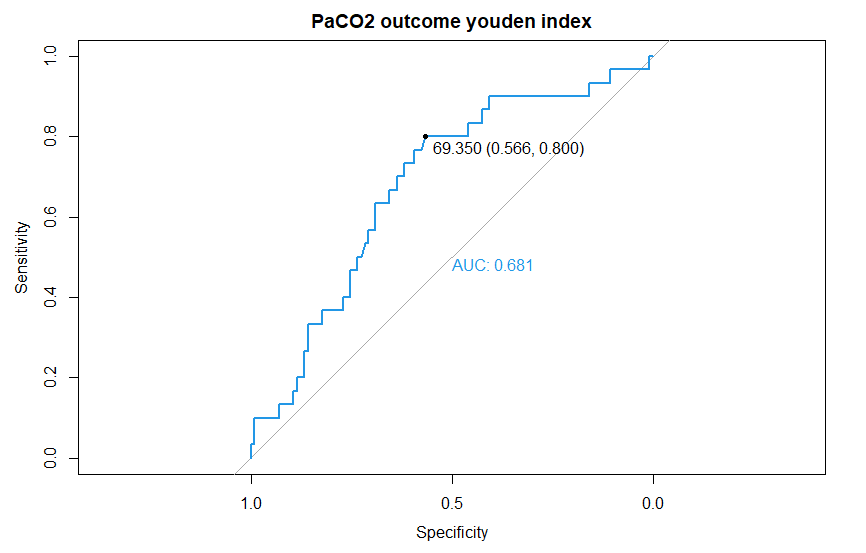


| PaCO_2_ (mmHg) | sensitivity | specificity | Youden index |
| --- | --- | --- | --- |
| 69.35 | 0.566 | 0.800 | 1.366 |
| 67.30 | 0.593 | 0.767 | 1.360 |
| 69.70 | 0.558 | 0.800 | 1.358 |
| 63.20 | 0.619 | 0.733 | 1.353 |
| 68.10 | 0.584 | 0.767 | 1.351 |
| 70.10 | 0.549 | 0.800 | 1.349 |
| 63.75 | 0.611 | 0.733 | 1.344 |
| 68.95 | 0.575 | 0.767 | 1.342 |
| 70.50 | 0.540 | 0.800 | 1.340 |
| 62.75 | 0.637 | 0.700 | 1.337 |
| 64.90 | 0.602 | 0.733 | 1.335 |

**Supplementary table 1. ROC curve and Youden index of PaCO_2_ and neurological outcome.**

| ECPR selection criteria of   1. CRITICAL study^1^: initial shockable rhythm + collapse-hospital time<45min + age <75yr 2. Lunz^2^: age≤65 years + witnessed cardiac arrest with bystander CPR + arrest-ECMO time <60min | |
| --- | --- |
| CRITICAL study | 100 patients meeting all criteria。 19 patients with FO (19%).  48 patients meeting all criteria + PaCO_2_ < 70mmHg。 16 patients with FO (33%). |
|  | 3 patients with FO were excluded.  FO percentage increased from 19% to 33%. |
| LUNZ | 37 patients meeting all criteria。 11 patients with FO (29%).  23 patients meeting all criteria + PaCO_2_ < 70mmHg。 8 patients with FO (34%). |
|  | 3 patients with FO were excluded.  FO percentage increased from 29% to 34%. |

**Supplementary table 2. Analysis of the study population with pre-specified ECPR criteria and PaCO_2_ 70 mmHg.**

1. Okada Y, Irisawa T, Yamada T, et al. Clinical outcomes among out-of-hospital cardiac arrest patients treated by extracorporeal cardiopulmonary resuscitation: The CRITICAL study in Osaka. Resuscitation 2022;178:116-23.

2. Lunz D, Calabrò L, Belliato M, et al. Extracorporeal membrane oxygenation for refractory cardiac arrest: a retrospective multicenter study. Intensive care medicine 2020;46:973-82.
